# Tools for quantitative form description; an evaluation of different software packages for semi-landmark analysis

**DOI:** 10.7717/peerj.1417

**Published:** 2015-11-19

**Authors:** Léo Botton-Divet, Alexandra Houssaye, Anthony Herrel, Anne-Claire Fabre, Raphael Cornette

**Affiliations:** 1 UMR 7179, Mécadev, Muséum National d’Histoire Naturelle, Centre National de la Recherche Scientifique, Paris, France; 2 Université Denis Diderot (Paris VII), Paris, France; 3 Evolutionary Morphology of Vertebrates, Ghent University, Ghent, Belgium; 4 Animal Locomotion Laboratory, Department of Evolutionary Anthropology, Duke University, Durham, NC, USA; 5 UMR 7205, Institut de Systématique, Évolution, Biodiversité, Centre National de la Recherche Scientifique, Muséum National d’Histoire Naturelle, Université Pierre et Marie Curie, Ecole Publique des Hautes Études, Paris, France

**Keywords:** Geometric morphometrics, Sliding semi-landmark, Software comparison

## Abstract

The challenging complexity of biological structures has led to the development of several methods for quantitative analyses of form. Bones are shaped by the interaction of historical (phylogenetic), structural, and functional constrains. Consequently, bone shape has been investigated intensively in an evolutionary context. Geometric morphometric approaches allow the description of the shape of an object in all of its biological complexity. However, when biological objects present only few anatomical landmarks, sliding semi-landmarks may provide good descriptors of shape. The sliding procedure, mandatory for sliding semi-landmarks, requires several steps that may be time-consuming. We here compare the time required by two different software packages (‘Edgewarp’ and ‘Morpho’) for the same sliding task, and investigate potential differences in the results and biological interpretation. ‘Morpho’ is much faster than ‘Edgewarp,’ notably as a result of the greater computational power of the ‘Morpho’ software routines and the complexity of the ‘Edgewarp’ workflow. Morphospaces obtained using both software packages are similar and provide a consistent description of the biological variability. The principal differences between the two software packages are observed in areas characterized by abrupt changes in the bone topography. In summary, both software packages perform equally well in terms of the description of biological structures, yet differ in the simplicity of the workflow and time needed to perform the analyses.

## Introduction

Because the interaction of form and function impacts performance, biological shape is under direct selection. The shape of a bone is influenced by several parameters. The evolutionary history of an organism plays an important role and often a strong phylogenetic signal is detected in bone shape (Morgan, 2009; Fabre et al., 2013a; Fabre et al., 2014a; Álvarez, Ercoli & Prevosti, 2013). Moreover, structural factors can shape bones as physical laws constrain potential shapes and their morphogenesis (e.g., Cubo, 2004; Cubo et al., 2008). For example, functional constraints imposed through differences in locomotor behavior (e.g., Fabre et al., 2013b; Álvarez, Ercoli & Prevosti, 2013) and prey capture strategy or prey size (Andersson, 2004; Meloro et al., 2008; Cornette et al., 2013) can affect bone size and shape. Additionally, mechanical loads during development (e.g., Beaupre, Orr & Carter, 1990; Bass et al., 2002) and during the lifetime of an individual (Lanyon et al., 1982; Currey, 2003) can shape bones because of bone remodeling.

Morphometric approaches have been used extensively for quantifying shape variation in biological objects including bones (Adams, Rohlf & Slice, 2004). The first paradigm, often referred to as ‘traditional morphometrics’ was based on a statistical treatment of distances or ratios (Marcus, 1990). Yet, this approach is limited as the information captured by linear measurements does not describe the whole geometry. As a consequence, the visualization of the geometry subsequent to statistical analysis is not possible and objects with different shapes can theoretically give rise to similar measurements. Additionally, many structures are difficult to measure in practice, leading to the use of measures such as maximal length which are functionally but not anatomically homologous.

These limitations have driven the development of geometric morphometrics in the 1990’s (Bookstein, 1991; Dryden & Mardia, 1993; Adams, Rohlf & Slice, 2004; Zelditch, Swiderski & Sheets, 2012). Geometric morphometrics use outline descriptors (Fourier analysis) or point coordinates (called landmarks) rather than distances to describe the geometry of biological objects. In this approach, size is explicitly defined (centroid size, sum of the squared distances of each landmark to the centroid; Bookstein, 1991) and the relative position of landmarks is conserved (Adams, Rohlf & Slice, 2004; Zelditch, Swiderski & Sheets, 2012).

Since a big part of biological variability cannot be assessed by using anatomical landmarks (biologically homologous landmarks) only, sliding semi-landmarks were developed to quantify complex shapes devoid of landmarks. Sliding semi-landmarks can be placed on curves (Bookstein, 1997; Gunz, Mitteroecker & Bookstein, 2005) and surfaces (Gunz, Mitteroecker & Bookstein, 2005). Due to the impossibility to define anatomically homologous points on curves and surfaces this approach generates landmarks that are spatially homologous after sliding (Parr et al., 2012). Sliding semi-landmarks are allowed to move on curves and surfaces in order to optimize a pre-defined criterion (Gunz & Mitteroecker, 2013). However, the choice of this criterion has been subject to debate. The most commonly used criterion is the bending energy (Skinner & Gunz, 2010; Pizzo et al., 2011; Cornette et al., 2013; Fabre et al., 2014b). The alternative involves the minimization of the Procrustes distance (Perez, Bernal & Gonzalez, 2006; Gunz & Mitteroecker, 2013). Sliding semi-landmarks are particularly well suited for the study of bones, providing descriptors of crests or outlines (curve sliding semi-landmarks; Morgan, 2009; De Groote, Lockwood & Aiello, 2010; Monteiro & Nogueira, 2010; Álvarez, Ercoli & Prevosti, 2013) and surfaces such as articular surfaces and the diaphysis of long bones (surface sliding semi-landmarks; Fabre et al., 2013a; Fabre et al., 2013b; Fabre et al., 2014b; Schlager, 2013a; Cornette et al., 2013; Morita et al., 2014; Cornette, Tresset & Herrel, 2014).

Several software packages are currently available that allow one to perform the sliding procedure in three dimensions. One of the first packages that was developed and that is still used in many studies (e.g., Kulemeyer et al., 2009; Fabre et al., 2013a; Fabre et al., 2013b; Cornette et al., 2013; Cornette, Tresset & Herrel, 2014) is ‘Edgewarp’ (Bookstein & Green, 1994). The ‘EVAN toolbox’ (http://evan-society.org) also performs sliding of semi-landmarks amongst other operations. Some authors have developed their own routines such as the Mathematica (Wolfram Inc., Modesto, California, USA) code used by Gunz & Mitteroecker (2013). Recently two R packages have been published: geomorph (Adams & Otárola-Castillo, 2013), featuring landmark placement, treatment and analysis, and ‘Morpho’ (Schlager, 2013b) featuring landmark importation from several other software packages and performing geometric morphometric treatment and analysis.

Because specimen digitization, the first step of any three dimensional surface analysis, is a time-consuming step that cannot be easily shortened, it could be of great interest to reduce the duration of the second step: the sliding procedure. The aim of the present study is to compare the workflow and the results obtained with two different software packages for the same three dimensional sliding task. We selected ‘Edgewarp’ (Bookstein & Green, 1994), an established reference for these types of analyses and ‘Morpho’ (Schlager, 2013b) a recently published set of R routines. We focused on a practical and biological test comparing a long bone in different mustelids. We followed the sliding procedure detailed in Gunz, Mitteroecker & Bookstein (2005) using both software packages. Differences between the results were examined and discussed in the light of the biological structures involved. The time required was measured for several steps and the global workflow (e.g., file handling, external software requirements) was compared.

## Materials and Methods

### Material

We used the humeri from 10 specimens belonging to five mustelid species (2 specimens per species; Table 1): *Meles meles* (Linnaeus, 1758), *Mustela putorius* (Linnaeus, 1758), *Gulo gulo* (Linnaeus, 1758), *Martes martes* (Linnaeus, 1758), and *Enhydra lutris* (Linnaeus, 1758). These species were chosen because they are widely distributed across the mustelid phylogeny and illustrate a relatively large diversity in both size, shape and ecology (Nowak, 2005; Schutz & Guralnick, 2007; Wilson et al., 2009; Hunter & Barrett, 2011). By choosing these specimens we attempted to encounter the largest number of potential pitfalls and difficulties during the sliding procedure. All specimens used are housed in the collections of ‘Mammiferes et Oiseaux’ from the Muséum National d’Histoire Naturelle in Paris, France.

**Table 1:** Specimens used in this study.

Species	Institution specimen
*Martes martes*	CG 1994-806
	CG 2005-232
*Gulo gulo*	CG 1983-946
	CG 1995-1208
*Mustela putorius*	CG 1991-605
	CG 2004-639
*Meles meles*	CG 2005-707
	CG 1987-28
*Enhydra lutris*	CG 1935-124
	A12503

**Notes.**

Institutional abbreviations are as follows CG Muséum National d’Histoire Naturelle Catalogue Général, Paris, France A Muséum National d’Histoire Naturelle Anatomie Comparée, Paris, France

### 3D modeling

Bones were digitized in three dimensions using a white light fringe Breuckmann 3D surface scanner (white light fringe StereoSCAN^3D^ model with a camera resolution of five megapixels). Raw scans were treated using Geomagic (Geomagic Studio; Raindrop Geomagic, Research Triangle Park, North Carolina, USA) to fill-in remaining holes and to remove highly creased edges and spikes. Next, models were decimated to contain 100,000 triangles resulting in homogeneous 3D surface models. The number of triangles was arbitrarily fixed to generate models that are not too cumbersome, but without altering the geometry of the object. The models were converted to ‘.sur’ using the ‘obj2sur’ tool provided at ‘ ftp://brainmap.stat.washington.edu/edgewarp/utils/’ in order to import them into ‘Edgewarp.’

### 3D anatomical landmark and curve digitization

**Figure 1:**
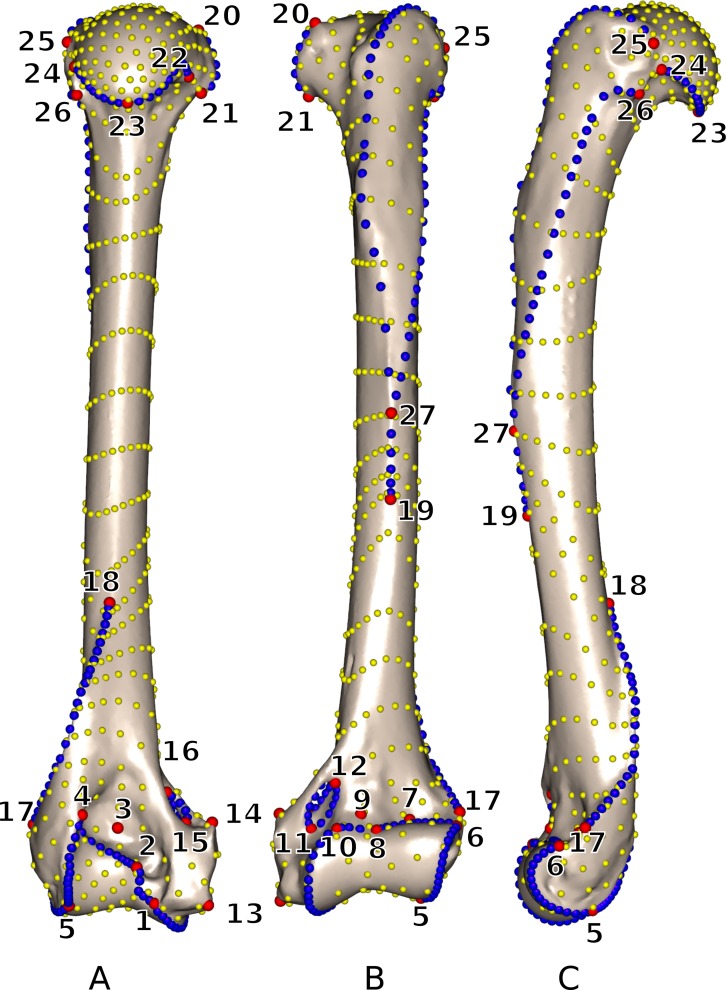
3D view of a *Martes martes* MNHN 2005-232 humerus showing the location of the 27 anatomical landmarks and 18 curves used to quantify the humeral shape. (A) caudal; (B) cranial; (C) lateral views. Red: anatomical landmarks. Blue: curve sliding semi-landmarks. Yellow: surface sliding semi-landmarks. See Table 2 for landmark definitions.

Landmarks and curves were digitized on the surfaces of the scans using the Landmark software package (Wiley et al., 2005). Twenty-seven 3D homologous anatomical landmarks were chosen and are visible on all specimens (Fig. 1 and Table 2). Eighteen 3D curves were defined at the margins of articular surfaces and along crests. All curves are bordered by anatomical landmarks as recommended by Gunz, Mitteroecker & Bookstein (2005). The curves were digitized with a high density of points (40–160 points per curve depending on the curve length) and then sub-sampled to the number listed in Table 3. This dataset was then used to perform an identical sliding procedure using both ‘Edgewarp’ and ‘Morpho.’

**Table 2:** Definition of the anatomical landmarks.

LM	Definition
1	Most disto-medial point of the trochlea
2	Most medio-proximal point of the caudal side of the trochlea
3	Point of maximum of curvature of the olecranon fossa
4	Most latero-proximal point of the caudal side of the trochlea
5	Most distal point of contact between the trochlea and the capitulum
6	Most latero-proximal point of the cranial side of the capitulum
7	Point of maximum of curvature of the radial fossa
8	Maximum concavity of the cranial margin of the trochlea
9	Point of maximum of curvature of the coronoid fossa
10	Most medio-proximal point of the cranial side of the trochlea
11	Most distal point of the cranial side of the supracondylar foramen
12	Most proximal side of the cranial side of the supracondylar foramen
13	Most distal tip of the medial epicondyle
14	Most proximal tip of the medial epicondyle
15	Most distal point of the caudal side of the supracondylar foramen
16	Most proximal point of the caudal side of the supracondylar foramen
17	Most disto-cranial point of the lateral epicondylar crest
18	Most proximal point of the lateral epicondylar crest
19	Most distal point of the deltopectoral crest
20	Upper tip of the lesser tuberosity
21	Most disto-medial point of the lesser tuberosity
22	Most medio-caudal point of contact between the lesser tuberosity and humeral head
23	Disto-caudal tip of the humeral head
24	Latero-caudal point of contact between the greater tuberosity and the humeral head
25	Most antero-proximal point of the greater tuberosity crest
26	Tip of the tuberosita teres minor
27	Contact point between tricipital line and greater trochanter crest

**Notes.**

LM landmark index

A semi-automatic point placement was used to place surface semi-landmarks on the scans. A template was modeled using the Blender software (Blender Online Community, 2014) following the procedure described in Souter et al. (2010). We created a mesh of 598 points representing a simplified form of the humerus. Landmarks and curves were digitized on the template surface as it was done on the actual scans of the specimens. Then in both ‘Edgewarp’ and ‘Morpho,’ landmarks and curves were used to compute a thin plate spline (TPS) deformation of the template. Next, surface sliding landmarks were projected from the deformed template onto the bone surface (Gunz & Mitteroecker, 2013). In summary, the template used in this study contains a total of 817 points including 27 3D homologous anatomical landmarks, 192 sliding semi-landmarks on curves and 598 sliding semi-landmarks on surfaces.

The sliding procedure was performed following the algorithm detailed in Gunz, Mitteroecker & Bookstein (2005). Four TPS relaxations were performed, the first TPS relaxation was performed against the template, the three others against Procrustes consensus calculated using the data from the previous iteration.

### Time estimation

The time for surface pre-processing and initial projection was measured by the value displayed in ‘Edgewarp’ log. The time required by ‘Edgewarp’ for the three iterations of sliding against Procrustes consensus was measured using a timer. The time required to run the ‘Morpho’ package in R was assessed by running the timestamp R function at the beginning and end of tasks and calculating the difference. These durations do not include time required for writing the script (scripts for data formatting for ‘Edgewarp’, and R scripting for ‘Morpho’). All analyses were run on a laptop computer (Asus k55vj) with a Intel^®^ i7-3630QM CPU, 4 Gb of memory, running on Linux Ubuntu V14.04.

### Geometric morphometrics and visualization

Data analysis and visualization were performed using the R software (R Core Team, 2014). In order to superimpose geometries and isolate size and shape, a generalized Procrustes analysis (GPA) was performed (Gower, 1975; Rohlf & Slice, 1990; Dryden & Mardia, 1998) for the ‘Edgewarp’ and the ‘Morpho’ dataset separately using the ‘Rmorph’ package (Baylac, 2013). We reduced dimensionality of the datasets by keeping the nine first non-null axes of a Principal Component Analysis (PCA) performed on the coordinates in the tangent space. In order to asses differences between the dataset produced by each software, a PROTEST analysis was performed using the ‘vegan’ package (Oksanen et al., 2015) run with 100,000 iterations (Peres-Neto & Jackson, 2001). The ‘vegan’ package was then used to draw the Procrustean superimposition plot. The three dimensional visualization of bone surfaces, landmarks, and vectors between homologous points were performed using the ‘rgl’ (Adler & Murdoch, 2012) and ‘Morpho’ (Schlager, 2013b) packages in R.

## Results

### Global shape variability

**Figure 2:**
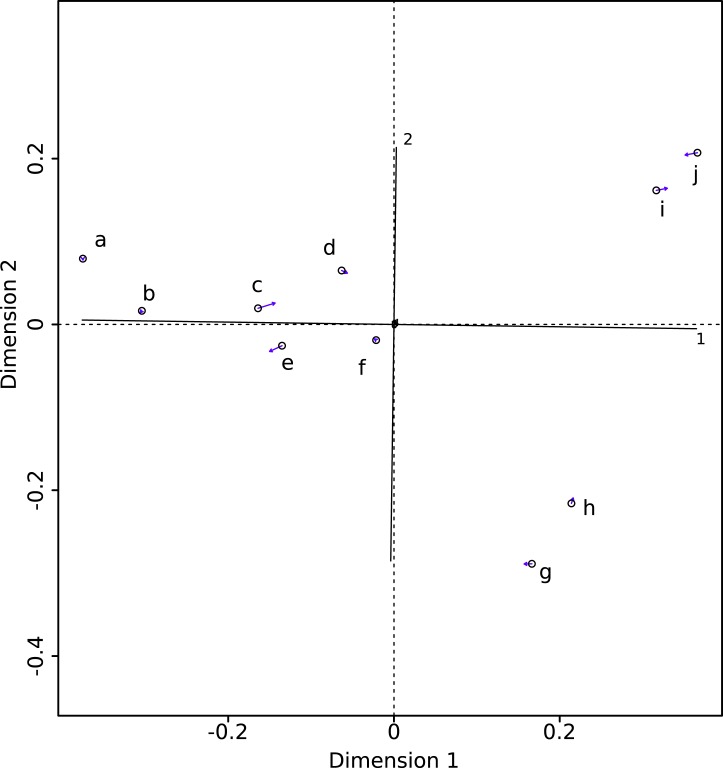
Procrustes superimposition of the Morpho (circles) and Edgewarp (arrow heads) results. Arrows present Procrustes residuals. (a) *Martes martes* 2005-232; (b) *Martes martes* 1994-806; (c) *Gulo gulo* 1983-946; (d) *Gulo gulo* 1995-1208; (e) *Mustela putorius* 1991-605; (f) *Mustela putorius* 2004-639; (g) *Meles meles* 2005-707; (h) *Meles meles* 1987-28; (i) *Enhydra lutris* 1935-124; (j) *Enhydra lutris* A12503.

The PROTEST analysis highly supports the congruence of the two datasets (*m*_12_ = 0.00325; *P* < 10^−5^). The Procrustean superimposition plot (Fig. 2) shows the differences between the two datasets. It becomes immediately clear that the between-methods variability is smaller than the inter-specimens variability, even within species. The Procrustes residuals of specimens analyzed with both methods vary from one specimen to the other (Table 4). The specimens showing the maximal Procrustes residuals are *Gulo gulo* 1983-946, *Mustela putorius* 1991-605, and *Enhydra lutris* 1935-124 (respectively c, e, and i; Fig. 2). The first axis separates *Enhydra lutris* (i and j; Fig. 2) and *Martes martes* (a and b; Fig. 2). On the two first axes *Mustela putorius* and *Gulo gulo* show intra-specific distances greater than the inter-specific ones with *Mustela putorius* 2004-639 (f; Fig. 2) being closer to *Gulo gulo* 1995-1208 (d; Fig. 2) than to *Mustela putorius* 1991-605 (e; Fig. 2) and *Gulo gulo* 1983-946 (c; Fig. 2) being closer to *Mustela putorius* 1991-605 (e; Fig. 2) than to *Gulo gul*o 1995-1208 (d; Fig. 2).

**Table 3:** Curve designation and number of sliding semi-landmarks per curve; for anatomical landmark designation please refer to Table 2 and Fig. 1.

Init LM	Term LM	NbSl
1	2	5
2	4	10
4	5	15
5	6	15
6	8	10
8	10	3
10	1	15
11	12	5
12	11	5
15	16	5
16	15	5
17	18	23
20	21	5
22	23	10
23	24	9
25	27	26
26	27	21
27	19	5

**Notes.**

Abbreviations are as follows init LM initial anatomical landmark index term LM terminal anatomical landmark index NbSl number of curve sliding semi-landmarks

**Table 4:** Procrustes residuals per specimen from the superimposition of the 9 first principal components of the Edgewarp and Morpho slid datasets.

Specimen	Procrustes residuals
*Enhydra lutris* 1935-124	0.020489693
*Enhydra lutris* A12503	0.017785627
*Gulo gulo* 1983-946	0.037204213
*Gulo gulo* 1995-1208	0.010347460
*Martes martes* 1994-806	0.009211963
*Martes martes* 2005-232	0.015334387
*Meles meles* 1987-28	0.008102059
*Meles meles* 2005-707	0.011307574
*Mustela putorius* 1991-605	0.021816036
*Mustela putorius* 2004-639	0.005777793

### Differences between mean shapes

**Figure 3:**
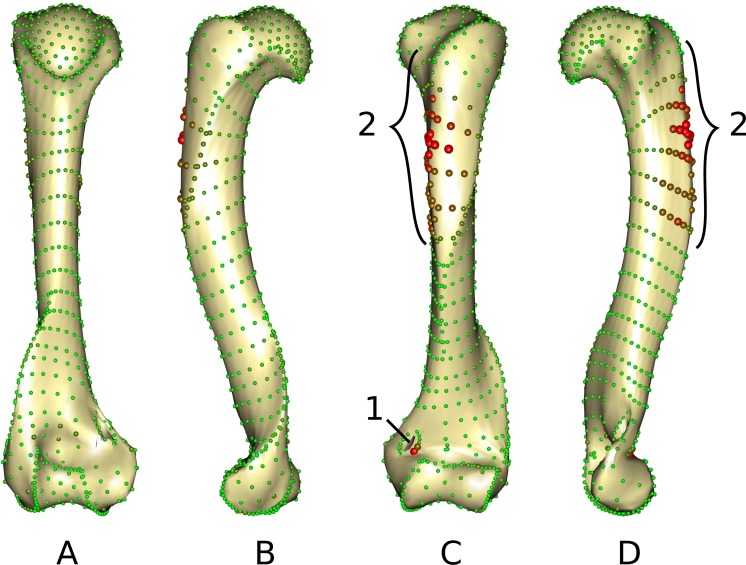
Warped mesh calculated on the Procrustes mean shape showing the most varying areas. (A) caudal; (B) lateral; (C) cranial; (D) medial views. Sphere colors (from green to red) and size are proportional to the Euclidean distance between points in the Procrustes mean shape for each software output after sliding. Points showing the maximal distance between the results obtained by the two software packages are large red points (as opposed to minimal distances being represented by small green points). 1: cranial side of the medial supracondylar foramen; 2: greater trochanteric crest.

The middle part of the greater trochanteric crest (2 in Fig. 3) presents the strongest differences. The neighboring points on the surface of the greater trochanteric crest are also affected. The second most variable area is the curve sliding around the cranial side of the medial supracondylar foramen (1 in Fig. 3) at the latero-distal part. Additionally, a point on the diaphysis located on the disto-medial side of the greater trochanter crest varies considerably.

### The most variable specimens

**Figure 4:**
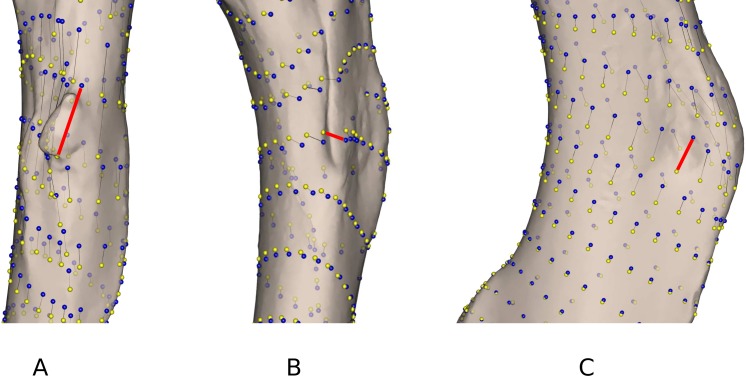
Close up view of the most variable areas of some specimens after sliding. Yellow: ‘Edgewarp’; blue: ‘Morpho’; black vectors link the homologous points in the output of each software package; the red vectors highlight the most variable points. (A) *Gulo gulo* 1983-946 medio-cranial view of the greater trochanteric crest; (B) *Mustela putorius* 1991-605 medio-caudal view of the lesser trochanter (distal) crest along the diaphysis. (C) *Enhydra lutris* 1935-124 medial view of the distal part of the greater trochanteric crest.

As highlighted in Fig. 2 and Table 4, differences linked to the software are more important in some specimens than in others. *Gulo gulo* 1983-946, *Mustela putorius* 1991-605, and *Enhydra lutris* 1935-124 display the largest differences between methods (Table 4). Beyond the differences noted in the mean shapes, we investigated the ones specific to each of these specimens. *Gulo gulo* 1983-946 (Figs. 4A) presents a displacement of points located on the deltoid crest, more specifically around the insertion of an ossified tendon (teres major/latissimus dorsi). The most variable point switches from one side of this structure to the other depending on the software used, suggesting that one of the methods did not succeed in sliding the point across this structure. *Mustela putorius* 1991-605 (Fig. 4B) presents a point shifting from one side to the other side of the crest on the diaphysis distal to the lesser trochanter. The ‘Edgewarp’ points (yellow) are located on the caudal side of the crest whereas the ‘Morpho’ points (blue) are located on the cranial side. *Enhydra lutris* 1935-124 (Fig. 4C) presents a variation associated with a small depression located medially to the deltoid crest. ‘Edgewarp’ points (yellow) slide on the distal margin of the foramen whereas the ‘Morpho’ points (blue) are close to the cranial margin of the depression. Surrounding points are also affected. The most variable points around the supracondylar foramen, at the latero-distal part of its cranial side are driven by the *Meles meles* 1987-28. For this specimen ‘Edgewarp’ points are concentrated on the most proximal part of this curve whereas ‘Morpho’ points are located more distally.

### Time

The times measured for surface pre-processing, the initial point projection, and the relaxations against the Procrustes mean shape, iterated three times, are given in Table 5. ‘Morpho’ requires a much shorter time compared to ‘Edgewarp’. The time required for the sliding against Procrustes mean shape by ‘Edgewarp’ is moreover an active time, meaning that the user needs to run each step of the iterations manually. This part also requires the computation of the GPA which is not implemented internally in ‘Edgewarp’. Based on the results of the GPA, new input files must be generated prior to performing the next iteration.

**Table 5:** Comparison of the duration of several tasks of the sliding procedure on the whole dataset with both ‘Edgewarp’ and ‘Morpho.’

	Edgewarp	Morpho	%
Surface pre-processing (s)	1,320	4	0.3
Initial point projection (s)	600	20	3.3
Sliding against mean shape 3 iterations (min)	105	2	1.9

**Notes.**

%, percentage of time required by ‘Morpho’ compared to ‘Edgewarp.’

## Discussion

### General variability

The distribution of specimens in morphological space is similar, irrespective of the software used. The biological information is similarly described with both packages and thus both can be used to describe complex biological shapes without an inherent bias. Nevertheless, as some local divergence between methods can be observed, results could differ for intra-specific studies often describing more subtle shape variations. The morphospace obtained after sliding is structured by the most extreme ecological niches in our sample suggesting that functional constraints drive much of the shape variability. The first dimension of the Procrustean superimposition plot separates *Enhydra lutris* and *Meles meles* (respectively i, j and g, h Fig. 2) from the other species. The maximal distance on this axis is obtained between *Enhydra lutris* (i and j, Fig. 2) which is the most aquatic of the Mustelidae sampled here (Estes, 1980; Bodkin, 2001) and *Martes martes* (a and b, Fig. 2) which is the most arboreal (Nowak, 2005) species in our sample. The second dimension of the Procrustean superimposition plot separates *Meles meles* (g and h, Fig. 2), the only semi-fossorial species in our sample (Nowak, 2005) from *Enhydra lutris* (i and j). Phylogeny, size and/or body mass (allometry) have been shown to be important factors shaping the humerus in Carnivora (Heinrich & Biknevicius, 1998; Fabre et al., 2013a; Martín-Serra, Figueirido & Palmqvist, 2014). Our data suggest that life-style is indeed an important driver of forelimb shape in mustelids. Nevertheless, this is a small sample only and these results should be interpreted with caution.

### Differences

As they are computed from the same dataset, morphospaces obtained by the two software where expected to be the same. The two morphospaces (relative distances between specimens) are indeed generally similar, which is validated by the significant PROTEST. Nevertheless some specimens show slightly different positions in the morphospace depending on the software (*Gulo gulo* 1983-946, *Mustela putorius* 1991-605, and *Enhydra lutris* 1935-124, respectively c, e, and i; Fig. 2). Specimens showing the greatest variation in their relative position between the two software are bones with areas where a structure interrupts the main curvature of the surface resulting in strong local deformations (Fig. 4). The fact that these local deformations are of a size similar to the distance between surface sliding landmarks appears to play a role. Indeed, highly curved structures can “puncture” the meshing constituted by the surface sliding semi-landmarks. Even if concerning only few points, these structures can lead to displacement of the specimen into the morphospace and could consequently have an impact in the biological interpretation of the morphospace (i.e., modifying phenetic affinities; Fig. 2). A greater number of surface sliding semi-landmarks in these specific areas could allow points to slide at the surface of a structure. Additionally, if the aforementioned structures present no biological meaning for the study, an a priori elimination could avoid giving too much weight to these structures in the analysis. Small foramina and ossified tendon insertions could be removed prior to the sliding procedure using dedicated tools in meshing software.

The differences observed between the two datasets thus likely result from differences during the sliding process at particular points of some specimens rather than reflect a global difference between methods. Such differences could be a consequence of differences between the two software packages in the computation of the relaxation as well as in the computation of the projection of the points on the surface of the specimen. Consequently mixing in a single analysis results obtained by the two software should be avoided.

### Time

‘Edgewarp’ appears more time-consuming than ‘Morpho’ for the same sliding task. ‘Edgewarp’ requires 30–300 times the time required by ‘Morpho’ for the same tasks. Two reasons can be put forward to explain these differences: computational efficiency and workflow complexity. ‘Edgewarp’ is a non-parallelized software whereas many ‘Morpho’ functions can be run on several cores, thus improving processing time. The effect of computation power remains low on a small dataset like the one presented here, but can lead to a considerable time difference in the case of the analysis of larger datasets. In addition to the number of steps constituting the workflow, the number of actions performed by the user should also be taken into consideration. The use of ‘Edgewarp’ requires one to open each surface, curve, and landmark file manually. The subsequent iterative relaxation requires several manual operations per iteration as does the data saving after sliding. The use of a programming language such as python (Van Rossum & De Boer, 1991), bash (Free Software Foundation, 2013), or even R (R Core Team, 2014) is useful to automate some parts of the workflow such as the ‘.sav’ generation. Once written, scripts can be run several times or adapted, reducing the time required to perform these repetitive tasks. Furthermore ‘Edgewarp’ does not perform the Procrustes superimposition needed for the computation of the mean shape, entailing the use of another software package (here R with the ‘Rmorph’ library). Therefore the time needed cannot be spent doing other things. Conversely using ‘Morpho,’ the principal active component is the writing of the script. Computation can then be run as a background task or on a remote computer. This is an advantage as much for an initial analysis of the data as for modifications (for example adding specimens or correcting a landmark placement).

### Problems encountered

Deviations from the initial curves were observed in ‘Morpho.’ This deviation from the initial curve is a logical consequence of the relaxation procedure along tangents. Surface sliding semi-landmarks tend to move inside or outside the volume of the specimen depending on the local curvature as they are sliding on the tangent (Gunz, Mitteroecker & Bookstein, 2005). Curve sliding landmarks move outside of the curve on the opposite side of the center of curvature. This is why points are projected back onto the surface after relaxation. But in some cases such as for the greater trochanteric crest (2 in Fig. 3) of *Gulo gulo* and *Martes martes* this leads to deviations of the curve from its initial position. This effect is counter balanced in ‘Edgewarp’ by re-projecting points onto the ‘rail.’ This ‘rail’ (named ‘.cur’ in ‘Edgewarp’) is constituted of a collection of points digitized on the bone surface, linked by segments. Consequently the definition of the ‘rail’ needs a large number of points to achieve a good description. However, when two curves are close, points can ‘jump’ from one curve to the other if the relaxed position is close to the second curve. Two strategies can counterbalance this effect in ‘Morpho’; first using a higher density of curve sliding landmarks (Gunz, Mitteroecker & Bookstein, 2005) and second, by reducing the relaxation step size. This last solution presents the advantage of being applicable on the initial dataset and does not require new data collection.

We also noticed points being projected on the wrong side of the supracondylar lateral crest and points projecting through bone from the olecranon fossa to the coronoid fossa while using ‘Edgewarp.’ The projection of surface sliding semi-landmarks from the template to the specimen surface is a crucial step preceding the actual sliding process. The main principle used in both software packages is a TPS deformation of the template constrained by anatomical and curve sliding landmarks followed by a projection onto the specimen surface. For some areas such as the lateral supracondylar crest, surface points can be projected onto the wrong side of the crest, if projected to the closest surface after TPS deformation. The ‘Morpho’ function ‘placePatch’ implements additional steps to avoid such effects. Inflation/deflation along the normals (vector giving the orientation of the mesh vertex) is performed; then the normals of projected points are compared to those of the template to avoid inside-out effects. In ‘Edgewarp,’ the graphical interface allows users to manually displace and correct wrongly projected points. The ‘Morpho’ strategy presents the advantage to be automated and integrated as a part of the ‘placePatch’ function. In contrast, ‘Edgewarp’ requires the manual displacement of the points, implying the correct identification of wrongly projected points prior to moving them to the correct side. This requires the investigation of all points on every specimen which is time consuming. The use of surface sliding semi-landmarks at the very vicinity of the curves appears to be a factor leading to projection on the wrong side of crests. Therefore, this problem could be circumvented by taking this fact into account during the design of the template.

Aside of the own properties of these software, the help file determines a part of the global ergonomy of any software package. Edgewarp’s user manual (User’s manual, EWSH3.19; available at ftp://brainmap.stat.washington.edu/edgewarp/) was last updated on March 2002. Additionally, it remains apparently unfinished as indicated by the author’s comments appearing along the text. On the other hand, Morpho’s help file is frequently updated and examples are provided for each function.

## Conclusions

This study shows that the results obtained by two software packages for 3D shape quantification, ‘Edgewarp’ (Bookstein & Green, 1994) and ‘Morpho’ (Schlager, 2013b), are similar and that biological variability is similarly described by both software packages. We highlight the fact that small structures jutting out of the surface as well as depressions may have an impact on the results and underline the importance of identifying these structures prior to the sliding operation. An increase in the density of the surface sliding landmarks in these areas or the removal of structures that do not contain biologically relevant information for the study are possible solutions for avoiding strong impact of these structures on the morphospace. The present study focuses on a small number of species, showing a large range of size and shape variation. This experimental design highlights the kind of structures that drive divergence between the two software packages. However, this design does not allow us to anticipate possible biases for different datasets such as those used in intra-specific studies where the variability between specimens is often quite subtle. Beyond the similarity in results, ‘Morpho’ is faster than ‘Edgewarp’ for the same sliding task, notably as a result of the computational power of ‘Morpho’ and the complexity of the workflow in ‘Edgewarp.’ ‘Edgewarp,’ on the other hand provides a visual approach to the sliding procedure. Thanks to the exploitation of modern computational power, ‘Morpho’ library, provides a powerful tool for the treatment of larger datasets.

## Supplemental Information

10.7717/peerj.1417/supp-1 Supplemental Information 1 Raw coordinates of anatomical and curve sliding semilandamarks for *Mustela putorius* MNHN 1991-605

10.7717/peerj.1417/supp-2 Supplemental Information 2 Raw coordinates of anatomical and curve sliding semilandamarks for *Mustela putorius* MNHN 2004-639

10.7717/peerj.1417/supp-3 Supplemental Information 3 Raw coordinates of anatomical and curve sliding semilandamarks for *Gulo gulo* MNHN 1995-1208

10.7717/peerj.1417/supp-4 Supplemental Information 4 Raw coordinates of anatomical and curve sliding semilandamarks for *Gulo gulo* MNHN 1987-28

10.7717/peerj.1417/supp-5 Supplemental Information 5 Raw coordinates of anatomical and curve sliding semilandamarks for *Gulo gulo* MNHN 1983-946

10.7717/peerj.1417/supp-6 Supplemental Information 6 Raw coordinates of anatomical and curve sliding semilandamarks for *Enhydra lutris* MNHN A12503

10.7717/peerj.1417/supp-7 Supplemental Information 7 Raw coordinates of anatomical and curve sliding semilandamarks for *Enhydra lutris* MNHN 1935-124

10.7717/peerj.1417/supp-8 Supplemental Information 8 Raw coordinates of anatomical and curve sliding semilandamarks for *Meles meles* MNHN 2005-707

10.7717/peerj.1417/supp-9 Supplemental Information 9 Raw coordinates of anatomical and curve sliding semilandamarks for *Martes martes* MNHN 1994-806

10.7717/peerj.1417/supp-10 Supplemental Information 10 Raw coordinates of anatomical and curve sliding semilandamarks for *Martes martes* MNHN 2005-232
